# High triglyceride-glucose index in young adulthood is associated with incident cardiovascular disease and mortality in later life: insight from the CARDIA study

**DOI:** 10.1186/s12933-022-01593-7

**Published:** 2022-08-12

**Authors:** Xinghao Xu, Rihua Huang, Yifen Lin, Yue Guo, Zhenyu Xiong, Xiangbin Zhong, Xiaomin Ye, Miaohong Li, Xiaodong Zhuang, Xinxue Liao

**Affiliations:** 1 Department of Cardiology, First Affiliated Hospital of Sun Yat-Sen University, 58 Zhongshan 2nd Road, Guangzhou, 510080 China; 2 NHC Key Laboratory of Assisted Circulation, (Sun Yat-Sen University), Guangzhou, China

**Keywords:** Triglyceride-glucose index, Cardiovascular disease, Mortality

## Abstract

**Background:**

This study aimed to investigate the associations between the triglyceride-glucose (TyG) index in young adulthood with incident cardiovascular disease (CVD) and mortality.

**Methods:**

We included 4,754 participants from the Coronary Artery Risk Development in Young Adults study at baseline. The TyG index was calculated as ln (fasting TG [mg/dl] × fasting glucose [mg/dl]/2), and the TyG index trajectories were identified by using the latent class growth mixture model. We evaluated the association between the baseline and trajectories of the TyG index with incident CVD events and all-cause mortality using Cox proportional hazards regression analysis. The added value of the TyG index included in pooled cohort equations for CVD prediction was also analyzed.

**Results:**

Among 4754 participants (mean age 24.72 years, 45.8% male, 51.2% black), there were 158 incident CVD events and 246 all-cause mortality during a median 25 years follow-up. After adjusting for multiple confounding variables, each one-unit increase in the TyG index was associated with a 96% higher CVD risk (hazard ratio [HR] 1.96, 95% confidence interval [CI] 1.44–2.66) and a 85% higher all-cause mortality risk (HR 1.85, 95% CI 1.45–2.36). Three distinct trajectories of the TyG index along the follow-up duration were identified: low (44.0%), moderate (45.5%), and high (10.5%). Compared with those participants in the low TyG index trajectory group, those in the high TyG index trajectory group had a greater risk of CVD events (HR 2.35, 95% CI 1.34–4.12) and all-cause mortality (HR 3.04, 95% CI 1.83–5.07). The addition of baseline TyG index to pooled cohort equations for CVD improved the C-statistics (P < 0.001), integrated discrimination improvement value (P < 0.001), and category-free net reclassification improvement value (P = 0.003).

**Conclusions:**

Higher baseline TyG index levels and higher long-term trajectory of TyG index during young adulthood were significantly associated with an increased risk of incident CVD events and all-cause mortality in later life.

**Supplementary Information:**

The online version contains supplementary material available at 10.1186/s12933-022-01593-7.

## Introduction

Cardiovascular disease (CVD) is the leading cause of global mortality and a major contributor to disability [1]. Over the past several decades, the incidence of CVD events has increased rapidly around the world [1] and tends to increase among younger individuals [2]. Therefore, early identifying those individuals at high risk of CVD and making effective preventive strategies for CVD starting at a young age is critically important. Insulin resistance is defined as a decrease in tissue response normally to insulin stimulation, which has been known to be one of the most critical risk factors for CVD [3, 4]. Recently, the triglyceride-glucose (TyG) index, measured by the fasting plasma glucose and triglyceride, has been proposed as a reliable surrogate marker of IR and shown to highly correlate with IR [5, 6]. Growing evidence has been demonstrated that the TyG index is related to adverse cardiovascular outcomes in the general population [7–9], as well as among certain high-risk patients, such as diabetes [10], hypertension [11], myocardial infarction [12], acute coronary syndrome [13], and after percutaneous coronary intervention [14]. In addition, several studies also report a predictive role of the TyG index with adverse cardiovascular outcomes among the general population [7, 15] and patients with cardiovascular diseases [12, 13]. However, most of the prior studies mainly focused on older adults with CVD events and mortality, and were inherently limited by short follow-up periods and the use of measured TyG index at a single time point. Data are still lacking regarding the associations between the TyG index and its trajectory derived from the multiple measurements over time with incident CVD events and mortality in younger, healthier adults.

Therefore, in this study, we analyze more than 25 years of longitudinal data from the Coronary Artery Risk Development in Young Adults (CARDIA) Study to assess the association between baseline TyG index and different trajectories of its change during young adulthood with CVD events and all-cause mortality in later life. Furthermore, we also evaluate the added value of the TyG index when included in the pooled cohort equations (PCEs) model in the prediction of CVD.

## Methods

### Study design and participants

The CARDIA study is a prospective cohort study that enrolled 5115 African American and white adults aged 18–30 years from four US field centers (Birmingham, Alabama; Chicago, Illinois; Minneapolis, Minnesota; and Oakland, California) from 1985 to 1986[16]. After the baseline examination (year 0), participants were invited to participate in follow-up examinations at years 2, 5, 7, 10, 15, 20, 25, and 30. Participants retention rates across these examinations were high: 91%, 86%, 81%, 79%, 74%, 72%, 72%, and 71%, of the surviving cohort, respectively. Additionally, semi-annual contact is maintained with participants via telephone, mail, or email, with annual interim medical history ascertainment; more than 90% of the surviving cohort participants have been directly contacted over the last 5 years. The institutional review board at all field centers approved the study protocols, and all participants provided written informed consent. All CARDIA data are obtained from the CARDIA Coordinating Center (https://www.cardia.dopm.uab.edu/contact-cardia). Details of the National Heart, Lung, and Blood Institute policies governing the data and how to access these data are available at (https://www.cardia.dopm.uab.edu/study-information/nhlbi-data-repository-data).

We exclude participants who had missing fasting glucose and triglyceride measurements at baseline (n = 101); those who had a history of heart problems at baseline (n = 306); and those who had missing data regarding other covariates of interest (n = 134). This resulted in a final sample of 4,574 participants to analyze the association between baseline TyG index with incident CVD events and all-cause mortality. We further excluded participants who had fewer than two valid TyG indexes during follow-up visits (n = 436); the remaining 4,138 participants were included in the analysis of the association between TyG index group-based trajectory with CVD events and all-cause mortality (Additional file 1: Figure S1).

### Data collection and definitions

At every CARDIA exam, information on participant demographics, anthropometrics, lifestyle, physical activity, biomarkers, family history, and use of medications were collected with standardized protocols [16]. At baseline in 1985, all participants reported age at enrollment, sex, race, and education level (total years achieved across follow-up in this study, for some participants were still pursuing education at baseline at age 18–30 years) on questionnaires. Serum glucose was measured at baseline using the hexokinase ultraviolet method developed by American Bio-Science Laboratories (Van Nuys, California, USA) and at examination years 7, 10, 15, 20, and 25 using hexokinase coupled to glucose-6-phosphate dehydrogenase (Merck Millipore, Billerica, Massachusetts). Before blood pressure assessment, participants were asked to fast and refrain from heavy physical activity for 5 min, and trained staff obtained 3 readings from the brachial artery. The mean of the second and third measurements was used for the analysis. Total cholesterol and triglyceride levels were assayed enzymatically within 6 weeks of fasting sample blood samples, and high-density lipoprotein cholesterol (HDL-C) was determined by precipitation with dextran sulfate-magnesium chloride. Low-density lipoprotein cholesterol (LDL-C) was calculated by the Friedewald equation [16, 17]. Body mass index was calculated as weight in kilograms divided by height in meters squared (kg/m^2^). Waist circumference (WC) was measured in duplicate by trained personnel, and the mean of two measurements was used for the analysis. Total physical activity during the past year was estimated from reports of the amount of time per week spent in 13 categories of physical activity and was calculated in exercise units [18]. Smoking status, alcohol consumption, and Medication use for hypertension were collected through interviewer-administered questionnaires. Hypertension was assessed using antihypertensive medication or SBP ≥ 140 mm Hg or DBP ≥ 90 mm Hg. Diabetes mellitus was defined at each examination by use of diabetic medication, fasting glucose ≥ 126 mg/dl (examination years 0 and 7–30), 2 h glucose ≥ 200 mg/dl (years 10, 20, and 25), or hemoglobin A1c (HbA1c) ≥ 6.5% (years 20 and 25) when available. The TyG index was calculated as ln(fasting TG [mg/dl] x fasting glucose [mg/dl]/2) [5].

### CVD and mortality outcomes

The primary outcome of interest was the first occurrence of any fatal or non-fatal CVD events, including myocardial infarction, non-myocardial infarction acute coronary syndrome, stroke, coronary revascularization, transient ischemic attack, congestive heart failure, carotid or peripheral arterial disease requiring intervention, or underlying cause of death due to CVD. All-cause mortality was examined as the secondary outcome. During annual phone interviews, people reported their own events and answered questions about hospitalizations. Deaths were identified by using an ongoing basis from family contacts and National Death Index queries; vital status follow-up of all participants is thus virtually complete. Two trained physician members of the Endpoints Committee independently reviewed medical records and adjudicated each possible cardiovascular or cerebrovascular event or underlying cause of death using standard definitions and a detailed manual of operations [19]. If disagreement occurred, the event was reviewed by the full committee. For this analysis, adjudication of events was complete through 2010–2011.

### Statistical analysis

Participants were stratified into quartiles according to their baseline calculated TyG index levels. Continuous data with normal distribution were presented as the mean ± SD and the skewed distribution data were presented as the median with interquartile ranges. Categorical data were presented as frequencies and percentages. The analysis of variance (ANOVA) or Kruskal–Wallis test was used to evaluate group difference for continuous variables, and the Pearson chi-square test was used for categorical variables.

The Kaplan–Meier method was used to compute the cumulative incidence of incident CVD events and all-cause mortality by TyG index quartiles, and differences among groups were compared using the log-rank test. Cox proportional hazards regression model was used to estimate hazard ratios (HR) and 95% confidence interval (CI) for the association between the TyG index with CVD events and all-cause mortality. Three multivariable models were built and used to adjust for potential confounders of CVD events and all-cause mortality. Model 1 was adjusted for sex, race, and age at baseline. Model 2 was additionally adjusted for body mass index (BMI), waist circumference (WC), systolic blood pressure (SBP), diastolic blood pressure (DBP), high-density lipoprotein (HDL-C), low-density lipoprotein (LDL-C), smoking status, drinking status, education level, and total physical activity. Model 3 was further adjusted for variables in model 2 plus diabetes, family history of diabetes, hypertension, and any antihypertensive use. The newly categorical variable was recorded as a continuous variable for the linear trend test and entered into the Cox proportional hazards regression models. We also analyzed the nonlinear dose–response association between baseline TyG index with incident CVD and all-cause mortality using a restricted cubic spline regression model with three knots. Subgroup analyses were stratified by baseline age (18–24 and 25–30 years), sex, race, total education level (up to/through high school and beyond high school), and BMI, respectively. In addition, we calculated the E-value to evaluate the robustness of the results to potential uncontrolled confounders [20].

Group-based trajectory modeling (GBTM) was designed to identify clusters of individuals following similar patterns of change over time [21]. We employed group-based trajectory modeling (GBTM) to identify different longitudinal TyG index level patterns over the entire follow-up and tested models with groups ranging from 2 to 5 [21, 22]. The optimal shape of trajectories (linear, quadratic, or cubic) and the number of groups were assessed by the Bayesian information criterion, with no group including less than 5% of the total participants and higher mean posterior probabilities (> 0.7). (Additional file 1: Table S2). Finally, three distinct trajectories turned out to be the best-fitting model. To evaluate the association of TyG index trajectory groups with incident CVD and all-cause mortality, the trajectory group was included as an independent variable in the Cox proportional hazards regression model examining predictors of incident CVD and all-cause mortality at follow-up.

To determine the optimal cutoff value of baseline TyG index in case of incident CVD and all-cause mortality, we used the receiver operating characteristic (ROC) curve analysis. The maximum value of the Youden index, calculated as sensitivity + specificity-1, was used to determine the appropriate cutoff point for the TyG index. Furthermore, we fitted a prediction model based on variables used in the pooled cohort equations (PCEs), including age, race, sex, total cholesterol, HDL-C, SBP, diabetes, treatment for hypertension, and smoking status. Then we developed a new model comprising the PCE variables with the addition of baseline TyG index and examine the added value of TyG index for CVD prediction in term of Harrell’s concordance statistic (C index), integrated discrimination index (IDI) and net reclassification index (NRI) [23].

All analyses were conducted in R version 4.1.3 (R Foundation for Statistical Computing, Vienna, Austria), SPSS version 25 (SPSS, Inc., Chicago, Illinois) and Stata 17.0 (Stata Corp LLC, Texas, USA). A two-sided P value < 0.05 was considered statistically significant.

## Results

### Baseline characteristics

Baseline characteristics of the 4,574 participants included in this study are shown in Table 1. The mean age of the total participants was 24.72 ± 3.62 years, 2,093(45.8%) were male, and 2,344 (51.2%) were black. The average baseline TyG index was 7.87 ± 0.52. We divided the participants into 4 groups based on the quartiles of the baseline TyG index. Participants with higher TyG index were more likely to be older, male, and white; they were more frequently to have a higher level of SBP, DBP, BMI, WC, TC, LDL-C, TG, and FPG; they were also a higher proportion of smoker, hypertension, and take antihypertensive medication, diabetes, and family history of diabetes; but similar for drinking, education level and total physical activity across the TyG quartiles.

**Table 1:** Baseline characteristics of participants according to the quartiles of baseline TyG index

	Total (n = 4,574)	Quartile (n = 1,144)	Quartile 2 (n = 1,143)	Quartile 3 (n = 1,143)	Quartile 4 (n = 1,144)	*p* value
TyG index	7.87 ± 0.52	7.26 ± 0.21	7.68 ± 0.09	7.99 ± 0.09	8.54 ± 0.38	< 0.001
Age, years	24.72 ± 3.62	24.44 ± 3.67	24.59 ± 3.64	24.61 ± 3.57	25.26 ± 3.55	< 0.001
Sex						
Male	2093 (45.8)	400 (35.0)	478 (41.8)	533 (46.6)	682 (59.6)	< 0.001
Female	2481 (54.2)	744 (65.0)	665 (58.2)	610 (53.4)	462 (40.4)	
Race						
Black	2344 (51.2)	689 (60.2)	621 (54.3)	564 (49.3)	470 (41.1)	< 0.001
White	2230 (48.8)	455 (39.8)	522 (45.7)	579 (50.7)	674 (58.9)	
SBP, mmHg	110.33 ± 10.90	107.94 ± 10.52	109.03 ± 10.07	110.92 ± 11.08	113.44 ± 11.08	< 0.001
DBP, mmHg	68.55 ± 9.58	67.21 ± 8.56	67.59 ± 9.42	69.10 ± 9.70	70.31 ± 10.25	< 0.001
BMI, kg/m^2^	24.46 ± 4.82	23.36 ± 4.19	23.78 ± 4.42	24.55 ± 4.91	26.14 ± 5.20	< 0.001
WC, cm	77.59 ± 10.82	73.64 ± 8.63	75.63 ± 9.69	77.83 ± 10.54	83.27 ± 11.71	< 0.001
Total Cholesterol, mg/dL	176.56 ± 33.55	163.91 ± 28.96	171.32 ± 30.00	179.89 ± 33.34	191.11 ± 35.29	< 0.001
HDL-c, mg/dL	53.06 ± 13.12	58.18 ± 12.49	55.72 ± 12.93	52.43 ± 12.38	45.92 ± 11.29	< 0.001
LDL-c, mg/dL	108.91 ± 31.32	98.24 ± 26.49	104.78 ± 28.34	113.01 ± 32.40	119.62 ± 33.30	< 0.001
TG, mg/dL	72.97 ± 48.37	37.48 ± 7.54	54.18 ± 6.93	72.27 ± 9.81	127.94 ± 67.24	< 0.001
FPG, mg/dL	82.43 ± 14.94	78.12 ± 7.65	80.90 ± 7.28	82.67 ± 9.35	88.03 ± 25.33	< 0.001
Drinking	3953 (86.4)	968 (84.6)	980 (85.7)	995 (87.1)	1010 (88.3)	0.06
Smoking	1974 (43.2)	405 (35.4)	504 (44.1)	492 (43)	573 (50.1)	< 0.001
Educational level, years	14.83 ± 2.77	14.84 ± 2.07	14.90 ± 3.27	14.94 ± 3.29	14.64 ± 2.18	0.054
Hypertension	407 (8.9)	88 (7.7)	71 (6.2)	102 (8.9)	146 (12.8)	< 0.001
Antihypertensive medication	101 (2.2)	15 (1.3)	19 (1.7)	24 (2.1)	43 (3.8)	< 0.001
Diabetes	49 (1.1)	6 (0.5)	5 (0.4)	12 (1.0)	26 (2.3)	< 0.001
Family history of diabetes	634 (13.9)	129 (11.3)	149 (13.0)	179 (15.7)	177 (15.5)	0.005
Total Physical activity, EU	421.43 ± 302.52	413.15 ± 305.23	416.40 ± 293.38	426.52 ± 305.82	429.64 ± 305.54	0.505

Data are shown as mean ± SD or n (%). Baseline characteristics of the 4,574 eligible participants from the CARDIA study, stratified by baseline triglyceride glucose index. There were significant between-group differences in most covariates considered

TyG triglyceride-glucose, *BMI* body mass index, *WC* waist circumference, *SBP* systolic blood pressure, *DBP* diastolic blood pressure, *FPG* fasting plasma glucose, *HDL-c* high-density lipoprotein cholesterol, *LDL-c* low-density lipoprotein cholesterol, *TG* triglyceride, *EU* exercise unit

### Association between baseline TyG index with cardiovascular disease and mortality

**Fig. 1:**
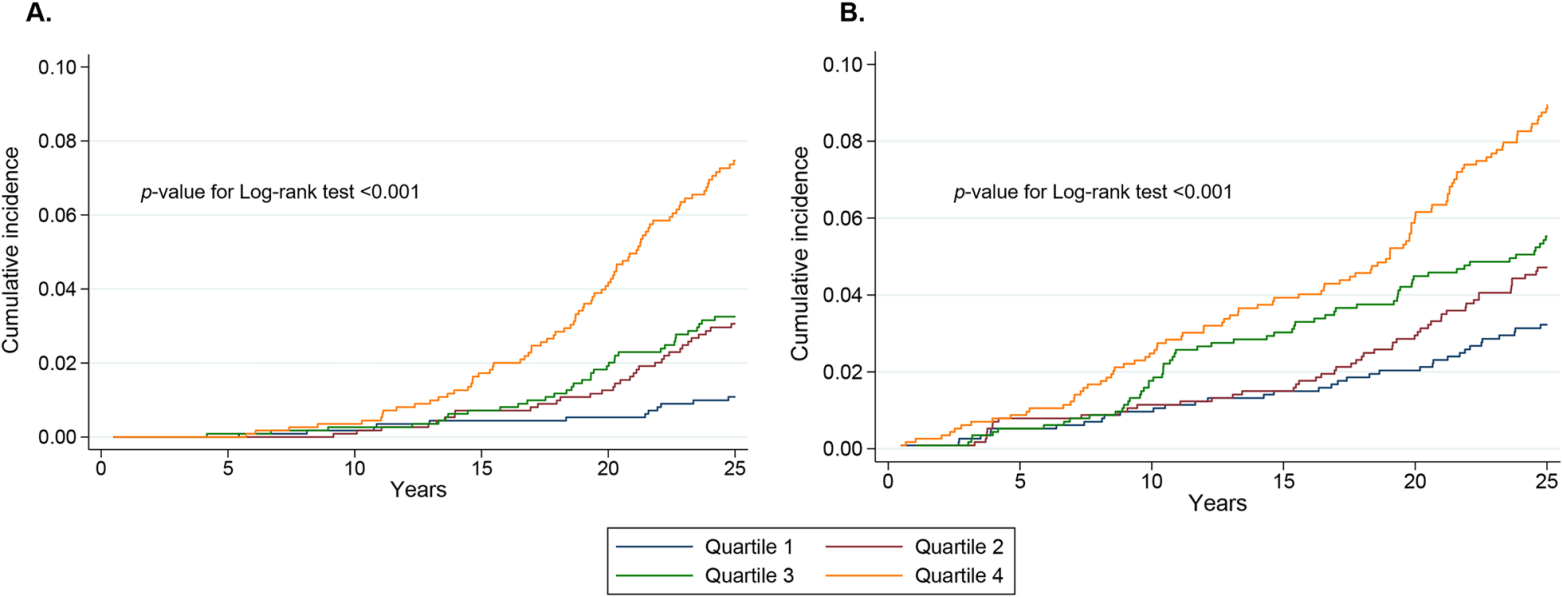
Cumulative incidence of cardiovascular disease (A) and all-cause mortality (B) by quartiles of baseline TyG index

During a median follow-up of 25 years, incident CVD events and all-cause mortality occurred in 158 and 246, respectively. Table 2 shows the Cox proportional hazard analysis of the association between the TyG index with incident CVD events and all-cause mortality. In the multivariate model that measured the TyG index as a continuous variable, per 1-unit increase in the TyG index was associated with a 96% higher risk of incident CVD, with a 85% higher risk of all-cause mortality after full adjustment for potential confounders in the model 3 (HR 85%, 95% CI 1.44–2.66, *p* < 0.001; HR 1.85, 95% CI 1.45–2.36, *p* < 0.001; respectively; Table 2). Similar results were shown when we categorized participants into quartiles by TyG index: participants with the highest levels of baseline TyG index had the highest risk of incident CVD and all-cause mortality in all different adjusted models (all P < 0.05, Table 2). After adjustment for potential confounding factors in model 3, compared to the reference, adjusted HRs (95% CI) for incident CVD for the second, third, and fourth quartiles were 2.41 (1.23–4.69), 2.19 (1.12–4.29), and 3.67 (1.90–7.06); and the corresponding HRs (95% CI) for all-cause mortality were 1.32 (0.86–2.03), 1.54 (1.00–2.35), and 2.18 (1.43–3.34), respectively (Table 2, Fig. 1).

**Table 2:** Risk of incident cardiovascular disease and mortality by baseline TyG index

	Model 1			Model 2		Model 3	
	Events/total	HR (95%CI)	*p* value	HR (95%CI)	*p* value	HR (95%CI)	*p* value
Cardiovascular disease							
Continuous (per unit)	158/4,574	3.20 (2.51, 4.09)	< 0.001	2.21 (1.63, 2.98)	< 0.001	1.96 (1.44, 2.66)	< 0.001
Quartiles							
Quartile 1 (≤ 7.5)	12/1,144	Reference	-	Reference	–	Reference	–
Quartile 2 (> 7.5, ≤ 7.8)	33/1,143	2.75 (1.42, 5.33)	0.002	2.33 (1.20, 4.54)	0.012	2.41 (1.23, 4.69)	0.009
Quartile 3 (> 7.8, ≤ 8.1)	35/1,143	3.06 (1.58, 5.91)	0.001	2.19 (1.12, 4.28)	0.021	2.19 (1.12, 4.29)	0.021
Quartile 4 (> 8.1)	78/1,144	6.73 (3.63, 12.47)	< 0.001	3.70 (1.92, 7.12)	< 0.001	3.67 (1.90, 7.06)	0.001
* p* for trend		< 0.001		< 0.001		< 0.001	
All-cause mortality							
Continuous (per unit)	246/4,574	2.12 (1.71, 2.61)	< 0.001	1.98 (1.56, 2.52)	< 0.001	1.85 (1.45, 2.36)	< 0.001
Quartiles							
Quartile 1 (≤ 7.5)	36/1,144	Reference	–	Reference	–	Reference	–
Quartile 2 (> 7.5, ≤ 7.8)	52/1,143	1.42 (0.93, 2.18)	0.103	1.34 (0.87, 2.05)	0.180	1.32 (0.86, 2.03)	0.193
Quartile 3 (> 7.8, ≤ 8.1)	61/1,143	1.68 (1.11, 2.55)	0.013	1.58 (1.03, 2.41)	0.032	1.54 (1.00, 2.35)	0.045
Quartile 4 (> 8.1)	97/1,144	2.56 (1.73, 3.79)	< 0.001	2.27 (1.48, 3.46)	< 0.001	2.18 (1.43, 3.34)	< 0.001
* p* for trend		< 0.001		< 0.001		< 0.001	

Model 1 Adjusted by sex, age, race

Model 2 Adjusted by model 1 + body mass index, waist circumference, systolic blood pressure, diastolic blood pressure, high-density lipoprotein cholesterol,low-density lipoprotein cholesterol, smoking status, drinking status, education level, total physical activity

Model 3 Adjusted by model 2 + diabetes, family history of diabetes, hypertension, any antihypertensive use

*TyG* triglyceride-glucose, *HR* hazard ratio, *CI* confidence interval

**Fig. 2:**
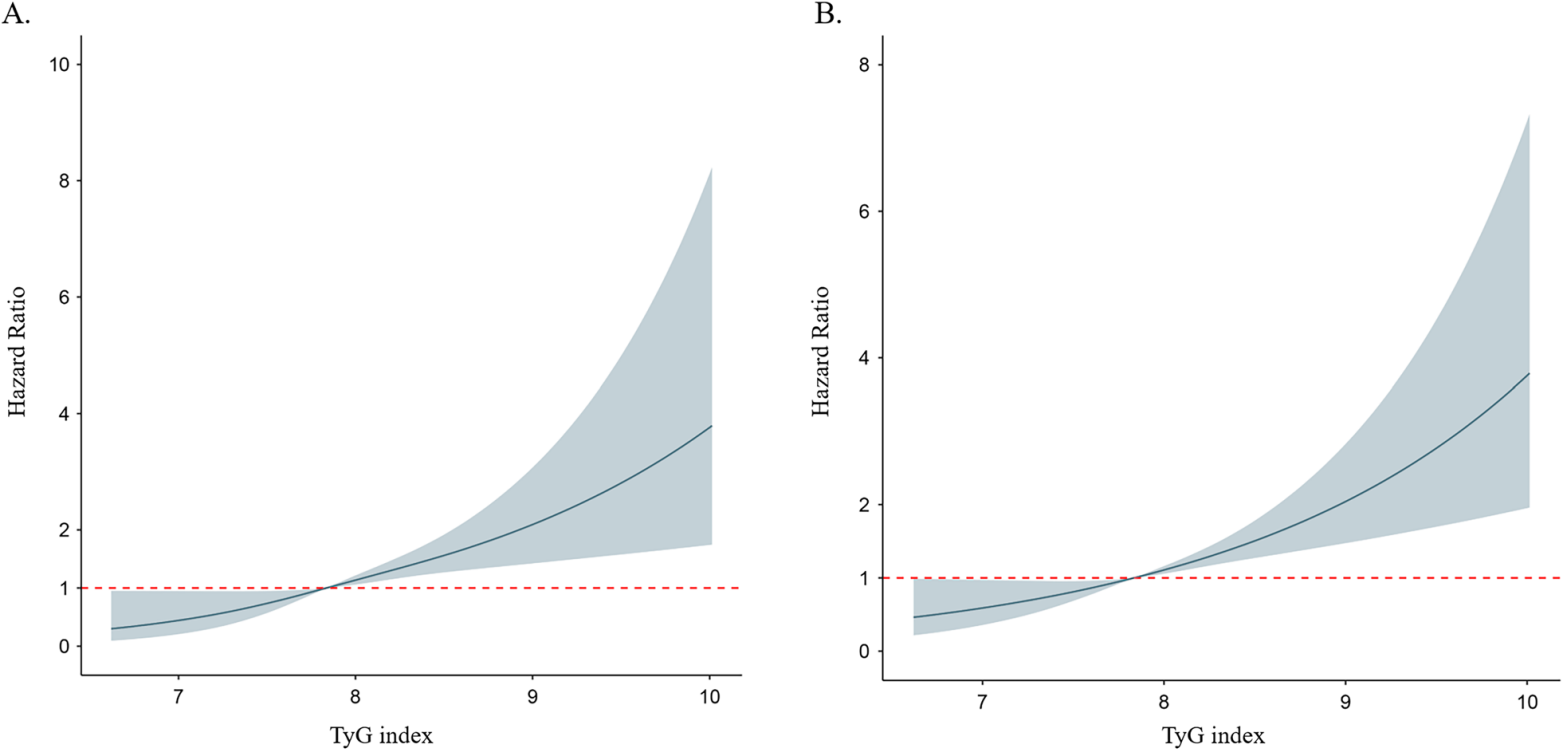
Adjusted hazard ratios of incident CVD events (A) and all-cause mortality (B) by baseline TyG index. Each hazard ratio was computed with a median baseline TyG index level of 7.8 (A and B). Both *p* for linearity < 0.01

**Fig. 3:**
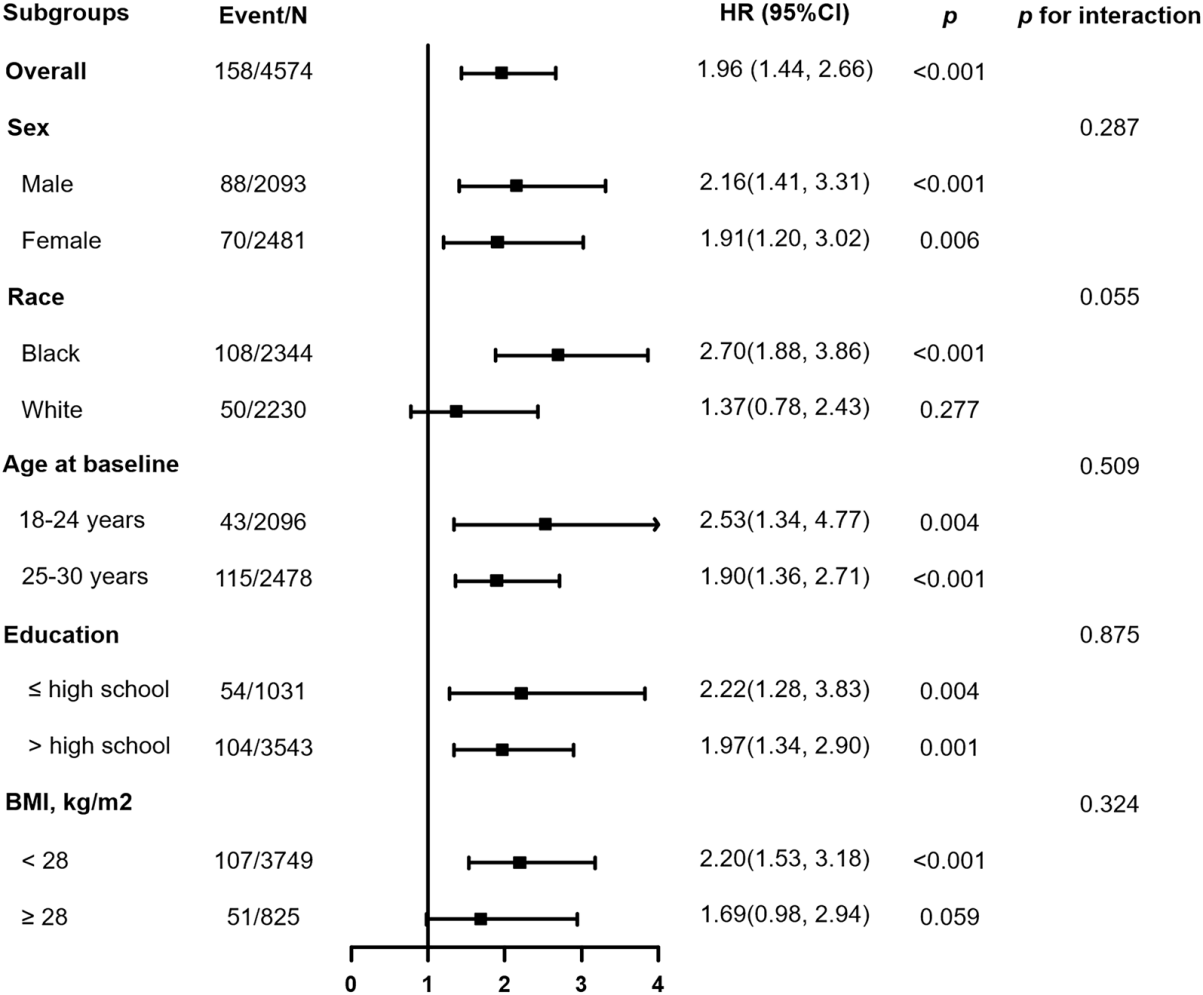
Subgroup analysis of the association between baseline TyG index and incident CVD events. Subgroup analysis included sex (male or female), race (black or white), age (≤ 24 or ≥ 25 years), education (≤ high school or > high school), and BMI (≤ 28 or > 28 kg/m^2^)

Multivariable adjusted restricted cubic splines regression models also showed linear associations between the baseline TyG index and the risk of CVD and all-cause mortality (Fig. 2). Increased TyG index (per 1-unit) was consistently related to CVD (Fig. 3) and all-cause mortality (Additional file 1: Figure S2) in various subgroups, including sex (male or female), race (black or white), age (≤ 24 or ≥ 25 years), education (≤ high school or > high school), and BMI (≤ 28 or > 28 kg/m^2^). There was no significant interaction in the subgroup (all *P* for interaction > 0.05). Furthermore, the E-values for the CVD and all-cause mortality were evaluated for the TyG index and compared to the HRs for the established cardiovascular risk factors for these outcomes (Additional file 1: Table S1). The comparison showed it would be unlikely that an unmeasured confounder exists that could account for the identified associations between the TyG index with CVD and all-cause mortality.

### Association between TyG index trajectories with cardiovascular disease and mortality

**Fig. 4:**
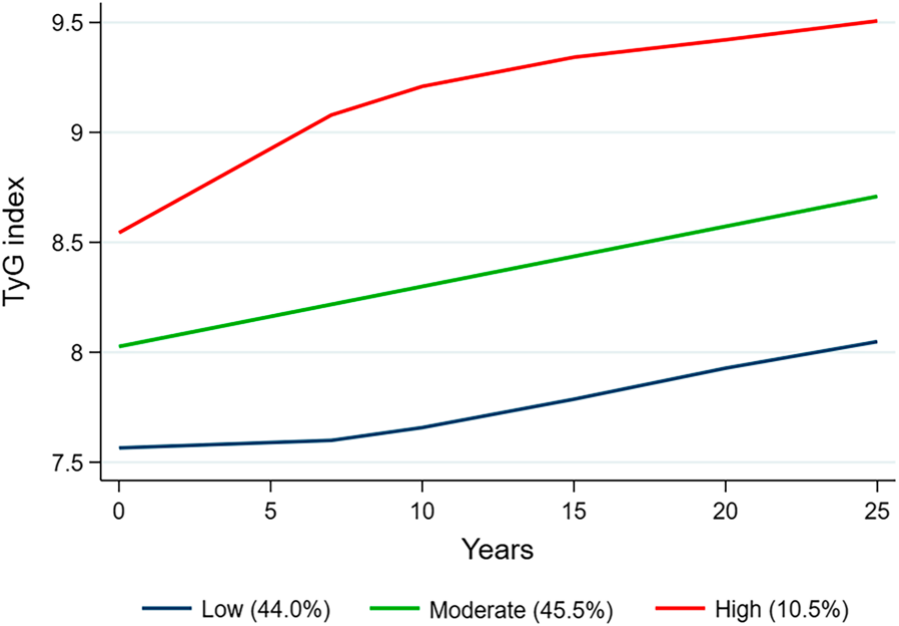
Trajectories by TyG index in the Coronary Artery Risk Development in Young Adults study

The trajectory analysis included a total of 4,138 participants finally. Three distinct TyG index trajectories were identified during the follow up (Fig. 4): low (n = 1,810, 44.0%), moderate (n = 1,910, 45.5%), and high (n = 418, 10.5%) TyG trajectory groups. During the follow-up, the median (interquartile range) changes between the final visit and baseline for these trajectory groups were 0.47 (− 0.01–0.95) in the low trajectory group, 0.67 (0.09–1.25) in the moderate trajectory group, and 0.97 (0.07–1.88) in the high trajectory groups (Additional file 1: Table S3). The incidence rate of CVD in the low, moderate, and high trajectory groups were 1.8%, 4.1%, and 8.9%; the corresponding rate of all-cause mortality were 2.5%, 5.0%, and 8.9%, respectively (Table 3). In the fully multivariate Cox regression model, taking the low group as a reference, adjusted HRs (95% CI) for associations of those participants with TyG index trajectories in the moderate and high groups with the risk of incident CVD were 1.58 (1.02–2.45) and 2.35 (1.34–4.12), and with the risk of all-cause mortality were 1.74 (1.19–2.53) and 3.04 (1.83–5.07), respectively (Table 3).

**Table 3:** Risk of incident cardiovascular disease and mortality by TyG index trajectory groups

	Model 1			Model 2		Model 3	
	Event/total (%)	HR (95%CI)	*p* value	HR (95%CI)	*p* value	HR (95%CI)	*p* value
Cardiovascular disease							
Low	32/1810 (1.8)	Reference	–	Reference	–	Reference	–
Moderate	79/1910 (4.1)	2.23 (1.47, 3.39)	< 0.001	1.58 (1.02, 2.45)	0.038	1.58 (1.02, 2.45)	0.039
High	37/418 (8.9)	4.88 (2.98, 7.98)	< 0.001	2.36 (1.35, 4.14)	0.002	2.35 (1.34, 4.12)	0.002
* p* for trend		< 0.001		0.001		0.005	
All-cause mortality							
Low	45/1810 (2.5)	Reference	–	Reference	–	Reference	–
Moderate	95/1910 (5.0)	1.87 (1.30, 2.69)	< 0.001	1.80 (1.23, 2.62)	0.002	1.74 (1.19, 2.53)	0.003
High	37/418 (8.9)	3.39 (2.15, 5.33)	< 0.001	3.21 (1.93, 5.33)	< 0.001	3.04 (1.83, 5.07)	< 0.001
* p* for trend		< 0.001		< 0.001		< 0.001	

Model 1 Adjusted by sex, age, race

Model 2 Adjusted by model 1 + body mass index, waist circumference, systolic blood pressure, diastolic blood pressure, high-density lipoprotein cholesterol, low-density lipoprotein cholesterol, smoking status, drinking status, education level, total physical activity

Model 3 Adjusted by model 2 + diabetes, family history of diabetes, hypertension, any antihypertensive use

*TyG* triglyceride-glucose, *HR* hazard ratio, *CI* confidence interval

### TyG index cut off points and added value of TyG index to PCEs model

The area under the curve (AUC) of the TyG index for incident CVD and all-cause mortality was 0.675, 95%CI (0.632–0.717) (Additional file 1: Figure S3A), and 0.612, 95%CI (0.575–0.648) (Additional file 1: Figure S3B), respectively. The cutoff points for the TyG index for incident CVD were 8.170 with 49.4% sensitivity and 76.6% specificity; the corresponding values for all-cause mortality were 8.025 with 49.2% sensitivity and 66.6% specificity, respectively.

The predictive value of the TyG index for CVD events was assessed using pooled cohort equations components (Table 4). The C index of the PCE model significantly improved after the addition of the TyG index (from 0.773 to 0.776, P < 0.001), and the discriminatory power and risk reclassification were also substantially better, with the IDI of 0.010 (95% CI, 0.004-0.017), and the NRI of 0.231 (95% CI,0.074–0.389).

**Table 4:** Performance of the PCEs model with TyG index to predict cardiovascular disease in the CARDIA study

	C-index		Category-free NRI		IDI	
	Index	*p* value	Index	*p* value	Index	*p* value
PCEs model	0.773 (0.738–0.808)		Ref		Ref	
PCEs model + TyG index	0.776 (0.742–0.810)	< 0.001	0.231 (0.074–0.389)	0.003	0.010 (0.004–0.017)	0.001

PCEs model: pooled cohort equations model, including age, race, sex, total cholesterol, high-density lipoprotein cholesterol, systolic blood pressure, diabetes, antihypertensive medication, smoking status

*TyG* triglyceride-glucose index, *NRI* net reclassification improvement, *IDI* integrated discrimination improvement

## Discussion

In this prospective cohort study, we found that a higher level of baseline TyG index in young adulthood was significantly associated with risks for incident CVD events and mortality in later life. In addition, we identified 3 distinct trajectories of the TyG index during follow-up, with the high trajectory group carrying the greater risk of future incident CVD events and mortality. Furthermore, the addition of the TyG index to the PCEs model significantly promoted the ability of risk stratification. These findings indicated that high levels of IR assessed by TyG index in late adolescence/young adulthood, as well as long-lasting high level of TyG index, is an important potential predictor and mediator for CVD events and all-cause mortality in later life.

Insulin resistance is an important risk factor for cardiovascular disease [3, 4]. The hyperinsulinemic-euglycemic clamp is the gold-standard test for IR assessment [24]; but is not frequently used in clinical practice and large population studies due to the complex detection process and economic reasons [25]. Recently, the TyG index, calculated using fasting blood glucose and triglyceride, has been reported to be a simple and reliable surrogate marker of IR and proved to correlate with the gold standard [6]. Several previous studies have reported that a high TyG index is related to the increased risk of cardiovascular disease and all-cause mortality in the general population. A recent meta-analysis included 5 studies for composite cardiovascular disease with 259,757 participants and found higher TyG index may be associated with an increased incidence of CVD in a linear association among the general population [26]. The pooled HRs (95% CI) for CVD among those with the highest TyG index was 1.46 (1.23–1.74) when compared with the lowest TyG index category. However, the studies included in this meta-analysis mainly focused on the middle-aged to the elderly population with the mean age range from 46.1 to 70.45 and based on the TyG index measured at a single time point [7–9, 27, 28], which may not capture long-term exposure due to the TyG index may vary over time. Therefore, the relationship between the TyG index and its longitudinal variation with cardiovascular outcomes in young populatoin needs to be evaluated. An analysis of the Atherosclerosis Risk in Communities (ARIC) Study [29], including 9097 participants in the analysis of TyG index trajectory groups and incident peripheral artery disease (PAD), have found that TyG index trajectories at highest levels had an greatest risk of future incident PAD. Thus, the measurement of the long-term trajectory of the TyG index could reflect the long-term impact of the TyG index on adverse health outcomes and provide more robust and reliable results. In line with the previous studies, our study found that the higher TyG index calculated at age 18–30 years was significantly associated with a higher risk of future CVD events. Moreover, our results revealed that those trajectory groups with long-term high TyG index levels beginning in young adult was at a higher risk of CVD events in mid-life. To the best of our knowledge, this is the first report concerning the impact of the long-term TyG index trajectory on the development of CVD events in the general population of young adults. In addition, the analysis of the E-value showed that it was unlikely that unmeasured confounding factors could eliminate the identified association between the TyG index and cardiovascular diseases in the present study. These findings demonstrate that higher baseline TyG index levels and higher long-term trajectory of TyG index during young adulthood were significantly associated with an increased risk of incident CVD events.

Another important finding of this study is that adding the TyG index to the pooled cohort equations (PCEs) model has an incremental effect on the predicted value of the CVD event. Previous studies have evaluated the predictive utility of the TyG index value for CVD prediction, but the results are not entirely consistent. An analysis of the Vascular Metabolic CUN cohort found that the addition of the TyG index to the Framingham model improves the areas under the receiver-operating characteristics curve from 0.708 to 0.719 [9]. Another study of Kailuan study also found that the addition of change in TyG to the conventional risk model had an incremental effect on the predictive value for incident CVD [15]. However, the analysis of the Tehran Lipid and Glucose Study reported that adding the TyG index to the Framingham risk score (FRS) did not improve its predictive power [7]. Racial differences among these studies may be an explanation for the discrepancies in the results. Finally, our study found that a higher baseline TyG index level in young adulthood is related to a higher risk of incident CVD events, and adding the TyG index to PCEs model can improve the predictive ability for CVD events, highlighting the usefulness of TyG index to early identify young individuals at high risk of developing a cardiovascular event.

Although the influence of the TyG index on adverse health outcomes has been receiving increasing attention, studies evaluating the relationship between the TyG index and mortality have been limited. In the recent meta-analysis of the TyG index and the risk of mortality in the general population [26], their results showed no association between the TyG index and mortality or all-cause mortality. In fact, among the four studies they included, two found a statistically significant association between the TyG index and all-cause mortality [30, 31], while the other two did not [28, 32]. In studies of different populations, most articles found a positive association between the TyG index and cardiovascular mortality/all-cause mortality [14, 33, 34]. Another recent study of the Kangbuk Samsung Health Study cohort [35], including 255,508 relatively healthy populations, supported that the TyG index is associated with an elevated risk of all-cause and cardiovascular mortality. Overall, in this present study, among the general population of young adults, the TyG index is significantly associated with a high risk of all-cause mortality, and the long-term TyG index trajectory analysis also obtained consistent results.

The potential mechanism underlying the association of IR assessed by the TyG index with incident CVD events and mortality is still uncertain; several speculations have been summarized as follows. Firstly, as a reliable surrogate index for IR, the TyG index is well reflected and closely related to IR, which can induce an imbalance in glucose and lipid metabolism, leading to chronic hyperglycemia and dyslipidemia. These metabolic changes have been reported as the causes of cardiovascular disease and all-cause mortality by epidemiological or genetic evidence [36, 37]. In addition, there is a close relationship between the TyG index and traditional risk factors for cardiovascular diseases such as obesity [38], diabetes [39], hypertension [40], and renal insufficiency [38, 41]. A high TyG index level is likely to reflect the adverse effects of impaired cardiometabolic health. Furthermore, IR has been found to have a strong relationship with endothelial dysfunction, cardiac metabolism changes, oxidative stress, and inflammation response [3, 4]. Thus, an elevated TyG index level may accelerate these processes and contribute to the development of cardiovascular diseases. Finally, the TyG index has been reported to be significantly associated with subclinical cardiovascular diseases, such as arterial stiffness [42, 43], carotid atherosclerosis [44, 45], and coronary artery calcification [46]. Therefore, an increase in the TyG index over time may lead to the process of cardiovascular diseases and mortality. Nevertheless, further studies are needed to clarify the precise role of the TyG index in cardiovascular diseases and mortality.

Our study has several important clinical implications for preventing the development of CVD events and mortality among the general population of young adults. First, in this current study, we have found a significant linear relationship between the TyG index with future CVD events and mortality, highlighting that the TyG index may serve as a valid predictor for identifying young individuals at high risk for developing CVD and mortality. Second, the analysis of the TyG index trajectories with CVD events and mortality provided reliable and robust results, indicating the usefulness of monitoring the long-term TyG index to identify high-risk individuals. Third, adding the TyG index to the PCEs model has an incremental effect on the prediction of CVD, demonstrating that the TyG index may improve the predictive power of existing cardiovascular risk scores in young adults. Finally, although the TyG index is not currently directly applied to clinical guidelines, the role of blood glucose and triglyceride control in preventing CVD is reflected in certain guidelines. The 2022 diabetes standard, recently released by the American Diabetes Association (ADA), states that patients with elevated triglyceride levels (150 mg/dl [1.7 mmol/l]) should implement enhanced lifestyle interventions and achieve optimal blood glucose control [47]. Therefore, preservation of an appropriate level of TG and FBG within the desirable range and taking better control of long-term TyG index into late adolescence or young adulthood are critically important for reducing adverse health outcomes in the future.

The key strengths of the current study include the use of its community-based prospective cohort design, a high proportion of Black participants, and a long follow-up of a unique age group. However, there are also several limitations in this study. First, due to the young baseline age of the population, this study has only captured the premature events in later life thus far. Second, only biracial black and white population were included in this study; results may differ in other ethnicities of this age range. Third, given the observational study design of the CARDIA study, the causal relationship between TyG index with CVD events and mortality could not be fully evaluated. Fourth, although multivariable has been adjusted in the Cox regression model, residual confounders were still possible, including the medical therapy throughout the follow-up, environmental and behavioral factors, etc. Fifth, the hyperinsulinemic-euglycemic clamp test was unavailable in this study, so we cannot assess the correlation between the TyG index with the gold standard of IR by the hyperinsulinemic-euglycemic clamp test.

## Conclusion

In summary, the current study shows that elevated levels of baseline TyG index and higher long-term trajectory of TyG index during young adulthood is strongly associated with high risk of incident CVD events and mortality in mid-life, independent of other traditional cardiovascular risk factors. Moreover, adding the TyG index to the PCEs model has an incremental effect on the predicted value of the CVD event. These findings support the contribution of a higher TyG index to the development of CVD events and mortality and indicate the importance of maintaining an appropriate level of TG and FBG within the desirable range beginning in late adolescence or young adulthood.

## Supplementary Information

**Additional file 1: Figure S1. **Flow chart for selecting the Coronary Artery Risk Development in Young Adults study participants for analysis. **Figure S2. **Subgroup analysis of the association between the baseline TyG index and all-cause mortality. **Figure S3. **The receiver operating characteristic (ROC) curves and diagnostic characteristics of the TyG index as a marker to predict CVD events (A) and all-cause mortality (B) in the CARDIA study. **Table S1. **E-value for the association between baseline TyG index with cardiovascular diseases and all-cause mortality (and its upper limit of 95% CI) in fully adjusted Cox models in CARDIA study. **Table S2. **Group-based trajectory model fit summary (N=4,138). **Table S3. **TyG index at examination years by trajectory groups of TyG index.

## Data Availability

All CARDIA data are obtained from the CARDIA Coordinating Center (https://www.cardia.dopm.uab.edu/contact-cardia). Details of the National Heart, Lung, and Blood Institute policies governing the data and how to access these data are available at (https://www.cardia.dopm.uab.edu/study-information/nhlbi-data-repository-data).

## Abbreviations

CARDIA: Coronary artery risk development in young adults
TyG: Triglyceride-glucose
CVD: Cardiovascular disease
SBP: Systolic blood pressure
DBP: Diastolic blood pressure
BMI: Body mass index
WC: Waist circumference
TC: Total cholesterol
TG: Triglyceride
LDL-c: Low-density lipoprotein cholesterol
HDL-c: High-density lipoprotein cholesterol
FBG: Fasting blood glucose
PCEs: Pooled cohort equations
