# Supplementary material for: High triglyceride-glucose index in young adulthood is associated with incident cardiovascular disease and mortality in later life: insight from the CARDIA study
Source: Cardiovasc Diabetol. 2022 Aug 12;21:155. doi: 10.1186/s12933-022-01593-7 (PMC9375240; doi:10.1186/s12933-022-01593-7)
Supplement: Supplementary file 1 — Additional file 1: Figure S1. Flow chart for selecting the Coronary Artery Risk Development in Young Adults study participants for analysis. Figure S2. Subgroup analysis of the association between the baseline TyG index and all-cause mortality. Figure S3. The receiver operating characteristic (ROC) curves and diagnostic characteristics of the TyG index as a marker to predict CVD events (A) and all-cause mortality (B) in the CARDIA study. Table S1. E-value for the association between baseline TyG index with cardiovascular diseases and all-cause mortality (and its upper limit of 95% CI) in fully adjusted Cox models in CARDIA study. Table S2. Group-based trajectory model fit summary (N=4,138). Table S3. TyG index at examination years by trajectory groups of TyG index. [file 12933_2022_1593_MOESM1_ESM.docx]

**High triglyceride-glucose index in young adulthood is associated with incident cardiovascular disease and mortality in later life: insight from the CARDIA Study**

Xinghao Xu^1,2†^, Rihua Huang^1,2†^, Yifen Lin^1,2†^, Yue Guo^1,2^, Zhenyu Xiong^1,2^, Xiangbin Zhong^1,2^, Xiaomin Ye^2^, Miaohong Li^1,2^, Xiaodong Zhuang^1,2*^ and Xinxue Liao^1,2*^

^1^ Department of Cardiology, the First Affiliated Hospital of Sun Yat-Sen University.

^2^ NHC Key Laboratory of Assisted Circulation (Sun Yat-Sen University).

*Supplemental Figure 1.* Flow chart for selecting the Coronary Artery Risk Development in Young Adults study participants for analysis

*Supplementary Figure 2.* Subgroup analysis of the association between the baseline TyG index and all-cause mortality.

*Supplementary Figure 3.* The receiver operating characteristic (ROC) curves of the TyG index as a marker to predict CVD events (A) and all-cause mortality (B) in participants in the CARDIA study.

*Supplementary Table 1*. E-value for the association between baseline TyG index with cardiovascular diseases and all-cause mortality (and its upper limit of 95% CI) in fully adjusted Cox models in CARDIA study

*Supplementary Table 2.* Group-based trajectory model fit summary

*Supplementary Table 3*. TyG index at examination years by trajectory groups of TyG index

**Additional file Figure S1. Flow chart for selecting the Coronary Artery Risk Development in Young Adults study participants for analysis**


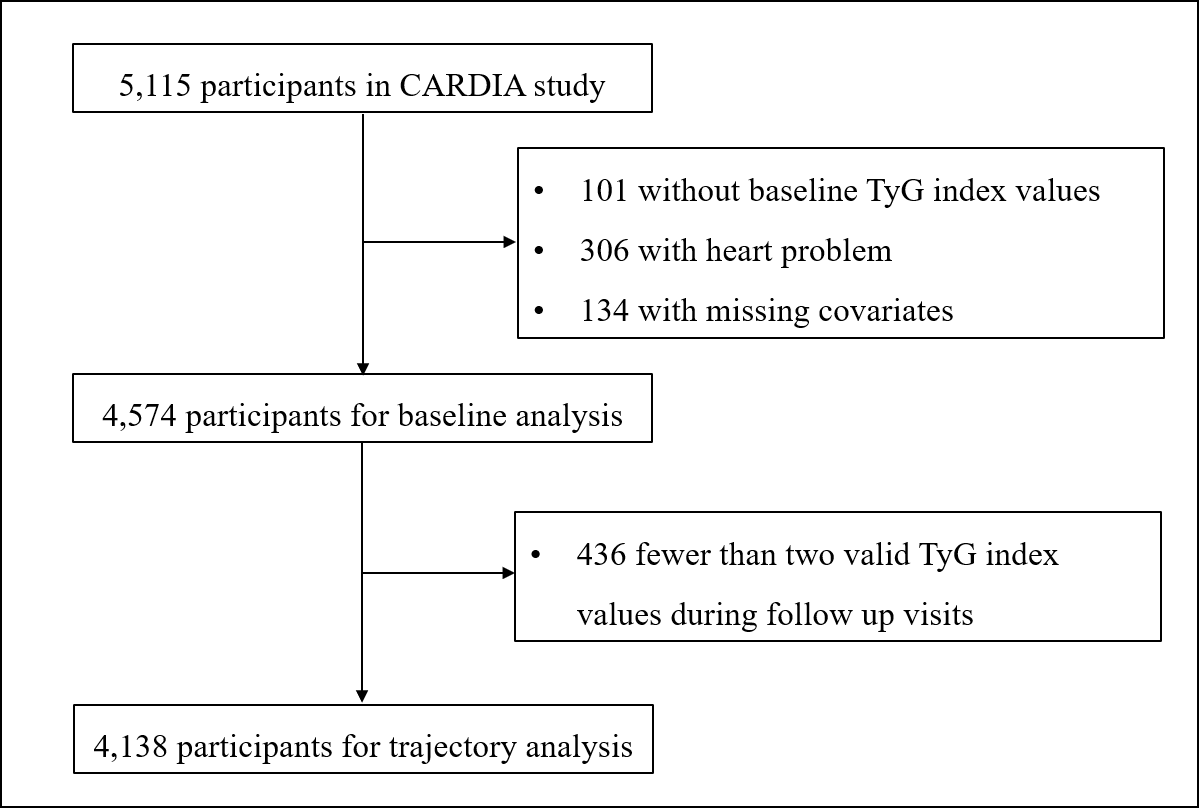


**Additional file Figure S2. Subgroup analysis of the association between the baseline TyG index and all-cause mortality.**


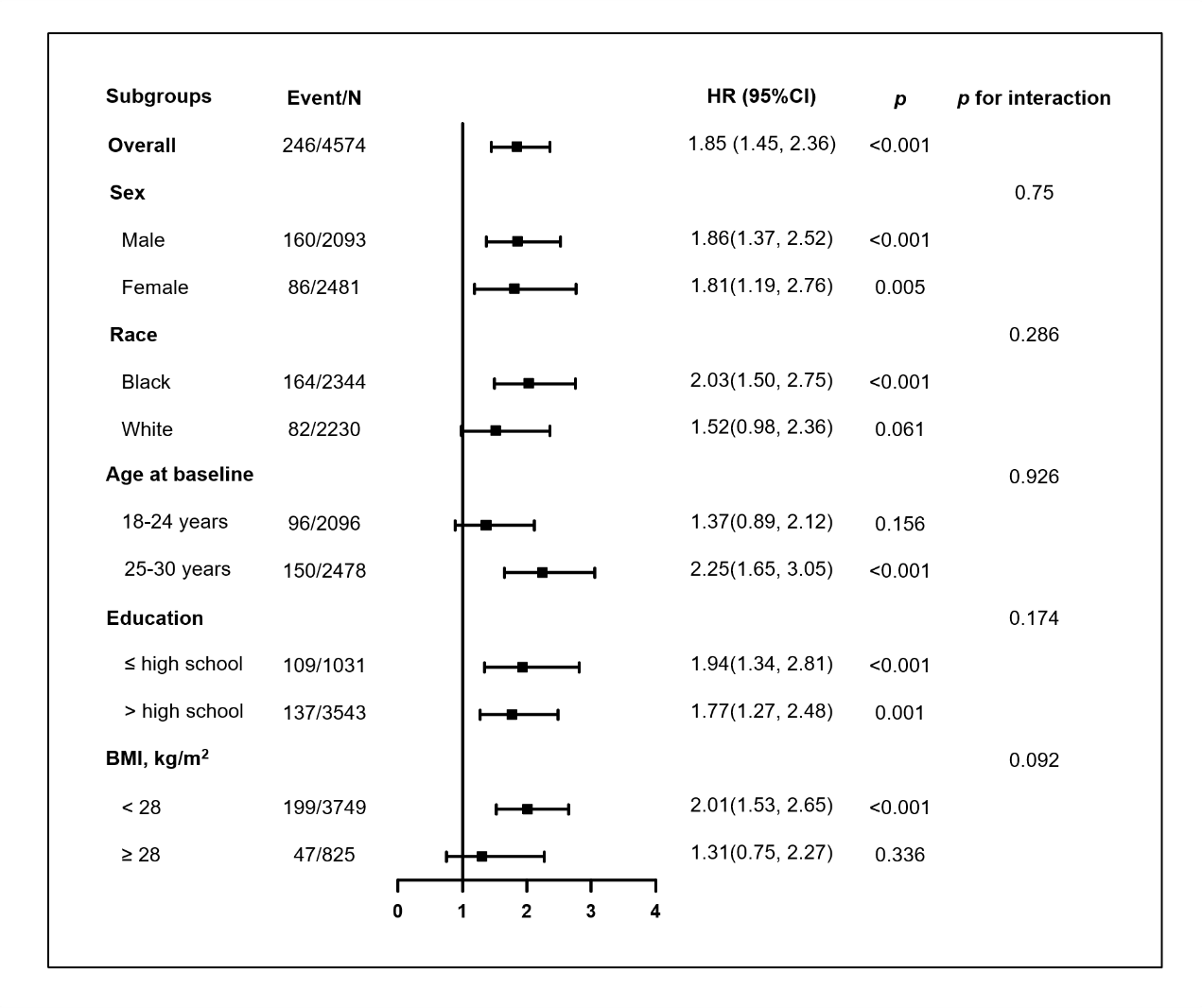


Subgroup analysis included sex (male or female), race (black or white), age (≤24 or ≥25 years), education (≤high school or > high school), and BMI (≤28 or >28 kg/m^2^).

**Additional file Figure S3. The receiver operating characteristic (ROC) curves and diagnostic characteristics of the TyG index as a marker to predict CVD events (A) and all-cause mortality (B) in the CARDIA study.**


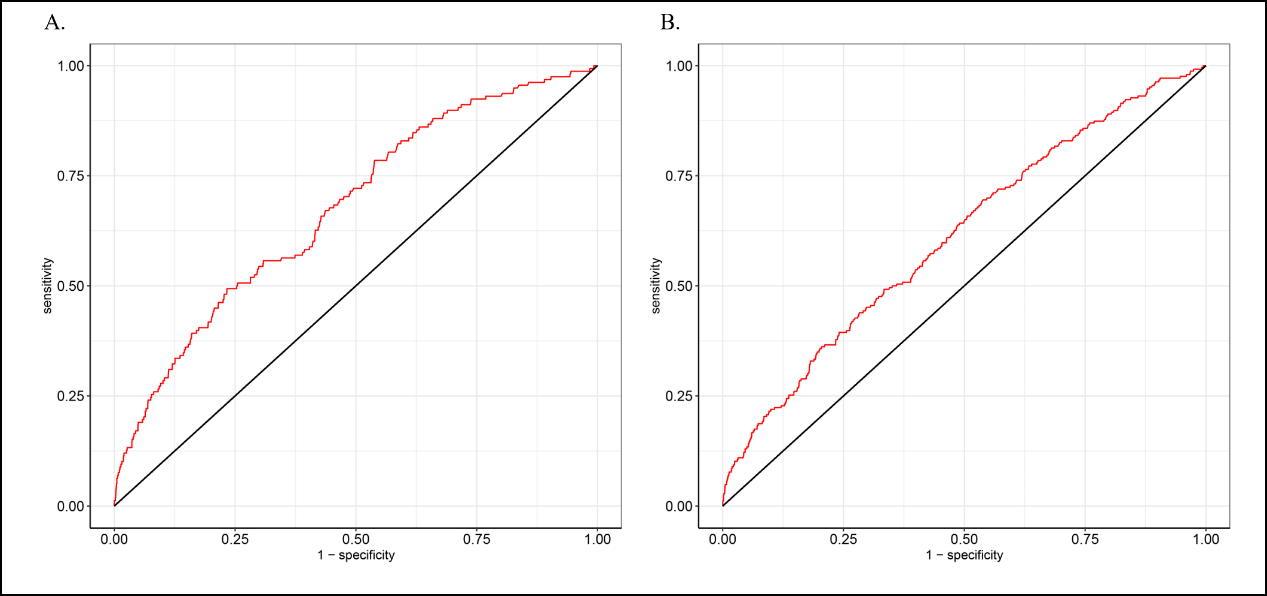


|  | AUC | Cut-off value | Sensitivity (%) | Specificity (%) |
| --- | --- | --- | --- | --- |
| CVD | 0.675(0.632-0.717) | 8.170 | 49.4% | 76.7% |
| All-cause mortality | 0.612(0.575-0.648) | 8.025 | 49.2% | 66.6% |

The area under the curve (AUC) of the TyG index for incident CVD and all-cause mortality were 0.675, 95%CI (0.632-0.717) (A) and 0.612, 95%CI (0.575-0.648) (B), respectively. The cut-off points for the TyG index for incident CVD was 8.170 with 49.4% sensitivity and 76.6% specificity; the corresponding values for all-cause mortality was 8.025 with 49.2% sensitivity and 66.6% specificity, respectively.

**Additional file Table S1.** E-value for the association between baseline TyG index with cardiovascular diseases and all-cause mortality (and its upper limit of 95% CI) in fully adjusted Cox models in CARDIA study.

| outcome | Fully adjusted HR (95% CI) for TyG index (per unit) | E-value for HR estimate | E-value for the lower limit of 95% CI | Variable | Level | HR |
| --- | --- | --- | --- | --- | --- | --- |
| CVD events | 1.96 (1.44, 2.66) | 3.33 | 2.24 | BMI | Per unit increased | 0.94 |
|  |  |  |  | WC | Per unit increased | 1.03 |
|  |  |  |  | Smoking | Yes vs. No | 1.84 |
|  |  |  |  | SBP | Per unit increased | 1.02 |
|  |  |  |  | LDL-c | Per unit increased | 1.01 |
| All-cause mortality | 1.85 (1.45, 2.36) | 3.10 | 2.26 | BMI | Per unit increased | 0.99 |
|  |  |  |  | WC | Per unit increased | 0.98 |
|  |  |  |  | Smoking | Yes vs. No | 1.71 |
|  |  |  |  | SBP | Per unit increased | 1.01 |
|  |  |  |  | LDL-c | Per unit increased | 1.00 |

**Additional file Table S2. Group-based trajectory model fit summary (N=4,138)**

| Model | AIC | BIC (N=20695) | BIC (N=4138) | Average posterior probability |
| --- | --- | --- | --- | --- |
| 2 | 15830.18 | 15865.90 | 15858.65 | 0.95/0.92 |
| 3 | 14570.23 | 14621.82 | 14611.36 | 0.92/0.89/0.92 |
| 4 | 14135.90 | 14199.40 | 14186.52 | 0.88/0.84/0.85/0.91 |
| 5 | 13942.87 | 14022.24 | 14006.15 | 0.86/0.81/0.82/0.87/0.94 |

*AIC* Akaike’s information criterion, *BIC* Bayesian information criterion.

**Additional file Table S3. TyG index at examination years by trajectory groups of TyG index**

|  |  | **TyG index trajectory groups** | | |
| --- | --- | --- | --- | --- |
|  |  | **Low**  **(n=1,810)** | **Moderate**  **(n=1,910)** | **High**  **(n=418)** |
| Years 0 | | 7.55 ± 0.37 | 8.01 ± 0.39 | 8.55± 0.58 |
| Years 7 | | 7.60 ± 0.38 | 8.26 ± 0.41 | 9.13 ± 0.64 |
| Years 10 | | 7.63 ± 0.37 | 8.32 ± 0.39 | 9.21 ± 0.58 |
| Years 15 | | 7.76 ± 0.34 | 8.43 ± 0.40 | 9.31 ± 0.59 |
| Years 20 | | 7.93 ± 0.35 | 8.63 ± 0.41 | 9.48 ± 0.60 |
| Years 25 | | 8.03 ± 0.36 | 8.67 ± 0.42 | 9.50 ± 0.66 |
| Change from Years 0 to Years 25 | | 0.47 (-0.01-0.95) | 0.67 (0.09-1.25) | 0.97 (0.07-1.88) |

*TyG* triglyceride-glucose.
